# Impact of Hepatitis C Seropositivity on the Risk of Coronary Heart Disease Events

**DOI:** 10.1016/j.amjcard.2014.09.020

**Published:** 2014-09-28

**Authors:** Naga Venkata Pothineni, Robert Delongchamp, Srikanth Vallurupalli, Zufeng Ding, Yao Dai, Curt H. Hagedorn, Jawahar L. Mehta

**Affiliations:** 1 Department of Medicine, University of Arkansas for Medical Sciences, Little Rock, Arkansas; 2 Department of Genetics, University of Arkansas for Medical Sciences, Little Rock, Arkansas; 3 Department of Epidemiology, University of Arkansas for Medical Sciences, Little Rock, Arkansas; 4 Central Arkansas Veterans Healthcare System, Little Rock, Arkansas

**Keywords:** Hepatitis C, Coronary Heart disease

## Abstract

Chronic infections have been shown to enhance atherogenicity. However, the association between chronic hepatitis C (HCV) and coronary heart disease (CHD) remains controversial. We examined the risk for CHD events in patients with HCV with an emphasis on the risk of CHD events with active infection. We conducted a retrospective cohort study using the Enterprise Data Warehouse at the University of Arkansas for Medical Sciences. HCV positive and negative patients were identified based on serology and incident CHD events were studied. Patient characteristics at entry were compared either by analysis of variance/F-test (continuous variables) or by a Chi-squared test (categorical variables). The joint effect of risk factors for incident CHD was evaluated using logistic regression. A total of 8,251 HCV antibody positive, 1,434 HCV RNA positive and 14,799 HCV negative patients were identified. HCV antibody and RNA positive patients had a higher incidence of hypertension, diabetes mellitus, obesity and chronic lung disease, but lower serum cholesterol levels compared to HCV negative patients (p< 0.001). HCV seropositive patients had a higher incidence of CHD events when compared to controls (4.9% vs. 3.2%, p<0.001). In the HCV cohort, patients with detectable HCV RNA had a significantly higher incidence of CHD events when compared to patients who were only HCV antibody positive with no detectable RNA (5.9% vs. 4.7%, p=0.04). In multivariate logistic regression analysis, both HCV antibody positivity (OR 1.32, 95% CI 1.09-1.60, p<0.001) and HCV RNA positivity (OR 1.59, 95% CI 1.13-2.26, p<0.001) were independent risk factors for incident CHD events. In conclusion, there is increased incidence of CHD events in HCV seropositive patients and the incidence is much higher in patients with detectable HCV RNA when compared to patients with remote infection who are only antibody positive. Lipid profile does not appear to be a good cardiovascular risk stratification tool in HVC patients.

## Introduction

A large body of evidence has linked chronic infections with atherosclerotic vascular disease. Transmission of infectious pathogens increases the extent of atherosclerosis in experimental animal models (1). Chronic infections have also been shown to increase the risk of coronary heart disease (CHD) events in humans (2, 3). However, an association between chronic hepatitis C (HCV) infection and cardiovascular risk has been supported by some (4-9), but not other studies (10-13). Some studies have even suggested that HCV infection may be protective against atherosclerosis (13). A recent systematic review suggested that the association between HCV infection and CHD events is inconclusive and needs additional research (14). Some of the reasons for discrepancy among these studies include the use of different diagnostic criteria for defining chronic HCV infection, small sample sizes, and use of different end-points. In addition, persistent infection with HCV (Ab+/RNA+) was not differentiated from remote HCV infection (Ab+/RNA-) that was cleared by host antiviral responses or by antiviral therapy. Using a large university based electronic medical records database, we sought to examine the effect of HCV infection on incident CHD events and to specifically study if patients with a detectable HCV RNA have a higher risk of CHD events.

## Methods

We conducted a retrospective cohort study using the Enterprise Data Warehouse (DW) at the University of Arkansas for Medical Sciences (UAMS). The DW is funded by the Translational Research Institute (TRI) at UAMS. The DW is updated monthly and maintains de-identified clinical information of nearly 1 million patients in the UAMS system. Information on patient demographics, international classification of diseases (ICD) 9 diagnoses, procedural codes, visit status, laboratory parameters and discharge disposition is available through the database.

We identified patients with a diagnosis of hepatitis C from January 1^st^ 2001 to December 31^st^ 2013. HCV infection was defined as the presence of HCV antibody as detected by enzyme-linked immunosorbent assay (ELISA) or a positive result of a qualitative or quantitative test for HCV RNA by polymerase chain reaction (PCR). Patients were divided into three study groups: a) patients with HCV antibodies by ELISA and no detectable HCV RNA by PCR in peripheral blood (Ab+/RNA-); b) patients with HCV antibodies and detectable HCV RNA (Ab+/RNA+); c) controls without HCV antibodies or RNA (Ab-/RNA-). The control group consisted of a randomly selected sex matched sample of HCV negative patients in the database within the study period. We labeled patients in group ‘a’ as having ‘remote’ HCV infection (previously treated or spontaneous clearance of infection or HCV RNA was never checked) and patients in group ‘b’ as having ‘persistent infection’. Patients in group ‘a’ and ‘b’ were all positive for HCV antibody, but patients in only group ‘b’ had positive HCV RNA. For patients in the HCV group, date of diagnosis of HCV (after January 1^st^ 2001) was chosen as the study initiation date. For HCV negative patients, date of first visit in the UAMS system after January 1^st^ 2001 was chosen as the study initiation date. The date of the last clinical visit since the study initiation date for all patients was selected as the study completion date. Validated International classification of diseases (ICD) 9 codes for hypertension, diabetes mellitus and chronic obstructive pulmonary disease (COPD) were used for identification of co-morbidities. CHD was defined by the presence of a diagnosis code for any of the following: coronary artery disease; chronic stable angina; unstable angina; or acute myocardial infarction. A CHD event was recorded if a patient had an ICD 9 code for any of the above diagnoses since the study initiation period. Patients who had multiple ICD 9 codes for CHD events mentioned above were counted as one CHD event. Obesity was defined as body mass index > 29.5 kg/m^2^. A diagnosis of COPD was used as a surrogate marker of smoking as actual smoking data in terms of pack years was not available. Patients in the HCV group who had a diagnosis of CHD (based on the above ICD9 code criteria) prior to their diagnosis of HCV were excluded from the study. Similarly, patients in the control group who had a diagnosis of CHD prior to the beginning of the study period were excluded.

Demographic data collected included age, sex and race. Laboratory data collected included HCV antibody status, quantitative HCV RNA by PCR, total cholesterol (T-C), low-density lipoprotein cholesterol (LDL-C), high-density lipoprotein cholesterol (HDL-C), triglycerides, alanine aminotransferase (ALT), aspartate aminotransferase (AST), gamma glutamyl transferase (GGT), alkaline phosphatase (ALP), serum albumin, total bilirubin, international normalized ratio (INR), hemoglobin A1c and serum creatinine. Patients' age at the study initiation period was used in the final analysis. Laboratory data available within the first 1 year of study initiation were collected. For patients who had repeat HCV serologies done, the first available HCV RNA PCR result was used for analysis.

Statistical analyses were computed with SAS version 9.4 (SAS Institute, Inc., Cary, NC). Patient characteristics at study initiation were compared among the three study groups either by analysis of variance/F-test (continuous characteristics) or by a Chi-squared test (categorical characteristics). The joint effect of risk factors for incident CHD was evaluated using logistic regression with odds ratios (OR) and their 95% confidence intervals reported as the estimate of risk. Differences in odds ratios for CHD among the three study HCV groups were also adjusted for all significant main effects and 2-way interactions among the set of CHD risk factors: age, sex, hypertension, COPD, diabetes and obesity.

## Results

Our final cohort consisted of 24,484 patients (8,251 HCV antibody positive, 1,434 HCV RNA positive and 14,799 HCV negative controls). Demographics of the patients are shown in Table 1. HCV antibody and RNA positive patients were significantly younger than control subjects. HCV seropositive patients had a significantly higher prevalence of traditional cardiovascular risk factors like hypertension, diabetes, obesity and COPD. These comorbidities were more frequent across both groups of HCV positive patients (antibody positive and RNA positive vs. Controls). Mean levels of T-C, LDL-C and HDL-C were significantly lower in the HCV cohort. As expected, HCV patients had significantly elevated levels of markers of liver injury (AST, ALT, GGT, ALP) when compared to HCV negative patients.

There were a total of 951 patients with documented CHD (per ICD codes), of which 471 were HCV positive and 480 were negative for HCV. There were 84 patients with CHD in the HCV RNA positive cohort and 387 patients in the HCV antibody positive cohort. HCV positive patients (groups a and b together) had a higher incidence of CHD events when compared to controls (4.9% vs. 3.2%, p<0.001). Furthermore, patients with detectable HCV RNA had a significantly higher incidence of CHD events when compared to patients who were only HCV antibody positive with no detectable RNA (5.9% vs. 4.7%, p=0.04).

In univariate analysis, age > 50 years, male sex, hypertension, diabetes, obesity, COPD and HCV Ab positivity and HCV RNA positivity were associated with an increased risk of incident CHD. After adjusting for age and sex, all the above conditions including HCV antibody and RNA positivity were significant risk factors for CHD (Table 2). In multivariate logistic regression analysis after adjusting for age, sex, hypertension, diabetes and COPD, HCV Ab and RNA positivity were independent risk factors for CHD. The odds ratio for developing a CHD event was 1.32 (1.09-1.59, p<0.001) in the HCV antibody positive group and 1.59 (1.13-2.26, p<0.001) in the HCV RNA positive group (Table 3). There was a significant interaction between hepatitis C positivity and obesity as competing risk factors for CHD in the hepatitis C positive population. Obese patients in the hepatitis C cohort had lower odds of incident CHD compared to non-obese hepatitis C positive patients (Table 3).

## Discussion

Hepatitis C is a common chronic viral infection that is estimated to infect 2% of people worldwide (15). At least 3.2 million adults in the United States have chronic HCV infection. Previous studies that examined the association of HCV infection and CHD risk have produced conflicting results, which may be due to several limits, including differences in diagnostic criteria, end points used and small sample sizes.

In our study of a large database from the state of Arkansas, we found an increased risk of CHD in HCV seropositive patients. HCV seropositivity was also an independent risk factor for CHD events. These results are in close parallel with the results reported by Butt et al (9). However, they reported the HCV patients to have a lower incidence of hypertension and diabetes. On the contrary, we found an increased incidence of these co-morbidities in our population sample. Interestingly, we found a significant interaction between HCV RNA positivity and obesity as competing risk factors. Obese HCV RNA positive patients had lower odds of CHD compared to non-obese HCV RNA positive patients. The reason for this interaction remains unclear. Presence or absence of obesity that was not examined in previous analyses could be a factor for the conflicting results in studies that examined the association of hepatitis C and CHD risk. Intriguingly, the “obesity paradox” is now being increasingly recognized in a wide range of patient populations. Studies have shown that patients with body mass indices (BMI) in the mild to moderately obese range have lower rates of cardiovascular mortality compared to patients with BMI in the underweight to normal range (16). We also found that HCV seropositive patients have relatively favorable baseline lipid profiles. This is in agreement with previous reports showing HCV positive patients to have lower lipid levels with an increase in lipid parameters with anti-viral therapy (17, 18).

A novel finding in our study is that patients with persistent hepatitis C infection, identified by a positive HCV RNA by PCR (Ab+/RNA+), had a much higher risk of CHD events than patients with remote infection that was cleared either spontaneously or by anti-viral therapy (Ab+/RNA-). Incidence of CHD events was 5.9% in the Ab+/RNA+ positive group compared to 4.7% in the HCV Ab+/RNA- group (p=0.04). To the best of our knowledge, this is the first study differentiating HCV antibody positivity and RNA positivity in terms of their relationship to CHD events. Studies have shown that active infections increase the pathogenicity of high fat diet in atherosclerosis-prone animals by increasing the uptake of oxidized LDL (2). It is possible that active infections illicit an immune response that induces plaque rupture in atherosclerotic arteries resulting in acute CHD events (19). The relationship between active infection and its impact on development of vascular events may also have therapeutic implications. Further studies are needed to confirm this observation and to determine if clearance of HCV with antiviral treatment alters the cardiovascular risk profile of patients.

Several pathophysiological mechanisms have been postulated to explain the association between chronic HCV infection and CHD. One is that chronic inflammation is a major pathognomic feature of atherosclerotic heart disease (20). Levels of inflammatory markers like IL-6, TNF-α and CRP are elevated in patients with chronic hepatitis C (21). Chronic inflammation in these patients may accelerate initiation and progression of atherosclerosis. Infection with HCV, in both cultured cells and patients, activates the NLRP3 inflammasome of macrophages and the production of IL-1β and other cytokines which may be the mechanism driving the higher incidence of CHD in patients with chronic hepatitis C (22). Alternative explanations for the association between chronic hepatitis C and CHD include: HCV induced insulin resistance and increased the risk of diabetes and metabolic syndrome which are traditional cardiovascular risk factors (23, 24); a higher incidence of substance abuse and smoking in patients with chronic hepatitis C patients which heighten CHD risk (13); or hepatic steatosis and non-alcoholic fatty liver disease that occurs in some patients with chronic hepatitis C has been reported to be pro-atherogenic (25).

Our study has several limitations. We examined a large population that receive regular care at a tertiary care teaching hospital and used appropriate ICD9 codes for comorbidities. We could not account for patients who were lost to follow up and may have received health care elsewhere. However, we think this could have equally affected both the HCV positive and HCV negative patient groups. Further, we could not adjust for the differences in medication usage, follow up visits, smoking status and family history of vascular disease between groups which could potentially alter the cardiovascular risk. The inclusion in study was dependent on ICD-9 CM codes, which are subject to inherent bias. However, selection of patients based on ICD codes has been shown to be associated with 94% sensitivity and 99% specificity (26). Lastly, there could have been some patients in the antibody positive group who were never tested for the presence of HCV RNA. These patients were included in the HCV RNA negative group in the analysis.

The strengths of the study, however, are a very large patient cohort followed at a single institution treated by same healthcare providers and followed for several years. The study at the onset identified patients with persistent infection and those with HCV antibody without ongoing infection.

## Figures and Tables

**Table 1:** Characteristics of the Patient Cohort

Characteristic	Hepatitis C Negative (n=14,799)	Hepatitis C Antibody Positive (n=8,251)	Hepatitis C RNA positive (n=1,434)	P value
Age (years)	53.0 ± 16.1	47.3 ± 10.9	48.6 ± 9.7	<0.001
Male	7992 (54.0 %)	4641 (56.3 %)	817 (57.0 %)	0.002
Female	6807 (45.9 %)	3610 (43.8 %)	617 (43.0 %)	0.002
White	8894 (60.1 %)	6287 (76.2 %)	1106 (77.1 %)	<0.001
African American	3507 (23.7 %)	1526 (18.5 %)	264 (18.4 %)	<0.001
Others	2398 (16.2 %)	438 (5.3 %)	64 (4.5 %)	<0.001
**Comorbidities**				
Diabetes Mellitus	755 (5.1%)	924 (11.2 %)	234 (16.3 %)	<0.001
Obesity*	2486 (16.8 %)	2385 (28.9 %)	641 (44.7 %)	<0.001
Chronic obstructive pulmonary disease	266 (1.8 %)	503 (6.1 %)	92 (6.4 %)	<0.001
Hypertension	1805 (12.2 %)	1898 (23.0 %)	443 (30.9 %)	<0.001
**Incident CHD events**				
Coronary Heart disease	480 (3.2 %)	387 (4.7 %)	84 (5.9 %)	<0.001
Liver Function**				
Albumin (mg/dl)	5.5 ± 4.3 (1985)	4.0 ± 2.6 (2485)	3.7 ± 1.4 (846)	<0.001
ALP (IU/L)	87.2 ± 56.3 (3455)	105.9 ± 88.7 (4257)	96.9 ± 48.3 (1203)	<0.001
ALT (IU/L)	37.5± 129.3 (3406)	74.0 ± 141.0 (4275)	91.0 ± 149.0 (1212)	<0.001
AST (IU/L)	40.7 ± 165.9 (3629)	88.0 ± 282.0 (4341)	88.8 ± 134.5 (1217)	<0.001
GGT (IU/L)	64.0 ± 128.8 (2989)	112.6 ± 165.1 (3822)	104.4 ± 137.6 (1017)	<0.001
Bilirubin (mg/dl)	0.9 ± 1.4 (3216)	1.7 ± 3.6 (4175)	1.4 ± 1.7 (1202)	<0.001
Lipid Profile**				
HDL-C (mg/dl)	47.3 ± 16.2 (2655)	42.6 ± 19.3 (1093)	43.0 ± 19.7 (226)	<0.001
LDL-C (mg/dl)	107.7 ± 50.9 (1995)	94.0 ± 40.6 (1968)	87.0 ± 35.9 (520)	<0.001
Cholesterol (mg/dl)	185.8 ± 49.7 (2858)	164.2 ± 63.0 (1169)	156.5 ± 55.7 (245)	<0.001
Triglycerides (mg/dl)	163.3 ± 329.8 (2802)	148.1 ± 178.0 (1323)	138.8 ± 105.0 (411)	0.10
Creatinine (mg/dl)	1.11 ± 0.95 (5440)	1.13 ± 1.16 (4429)	1.03 ± 1.09 (1163)	0.01
HbA1c (mg/dl)	6.9 ± 2.0 (1308)	7.0 ± 2.2 (602)	6.6 ± 2.2 (214)	0.06

CHD-Coronary heart disease; COPD-Chronic obstructive pulmonary disease; LDL-C-low-density lipoprotein cholesterol; HDL-C-high-density lipoprotein cholesterol; ALT-alanine aminotransferase; AST-aspartate aminotransferase; GGT- gamma glutamyl transferase; ALP-alkaline phosphatase;HbA1c – Hemoglobin A1c

* Obesity was defined as body mass index > 29.5

** Data presented as mean ± SD; Number of observations in parentheses

**Table 2:** Logistic Regression Analysis and Odds Ratio of Coronary Heart disease for Individual Risk Factors after Adjusting for Age and Sex

Risk Factor	Odds Ratio for CHD	95% CI	P value
Hypertension	4.84	4.23 – 5.53	<0.001
Obesity	2.98	2.60 – 3.41	<0.001
Diabetes	4.20	3.60 – 4.90	<0.001
Chronic Obstructive Pulmonary disease	3.66	2.97 – 4.51	<0.001
Hepatitis C (antibody positive)	1.67	1.45 – 1.91	<0.001
Hepatitis C (RNA positive)	1.95	1.53 – 2.50	<0.001

**Table 3:** Logistic Regression Analysis after Adjusting for All Significant Risk Factors (Age, Sex, Hypertension, Diabetes and Chronic Obstructive Pulmonary disease)

HCV status	Obesity	
	Yes	No
Negative	2.65 (2.07 – 3.38)	1*
Antibody positive	1.70 (1.31 – 2.19)	1.32 (1.09 – 1.60)
RNA positive	1.30 (0.78 – 2.16)	1.59 (1.13 – 2.26)

* reference group
